# Differential Expression Pattern of Goat Uterine Fluids Extracellular Vesicles miRNAs during Peri-Implantation

**DOI:** 10.3390/cells10092308

**Published:** 2021-09-03

**Authors:** Yanshe Xie, Guangbin Liu, Xupeng Zang, Qun Hu, Chen Zhou, Yaokun Li, Dewu Liu, Linjun Hong

**Affiliations:** 1 College of Animal Science, South China Agricultural University, Guangzhou 510642, China; xys@stu.scau.edu.cn (Y.X.); gbliu@scau.edu.cn (G.L.); xupeng_zang@stu.scau.edu.cn (X.Z.); qhu@stu.scau.edu.cn (Q.H.); czhou@stu.scau.edu.cn (C.Z.); ykli@scau.edu.cn (Y.L.); 2 National Local Joint Engineering Research Center of Livestock and Poutry, South China Agricultural University, Guangzhou 510642, China

**Keywords:** goat, uterine fluids, extracellular vesicles, miRNA, endometrium, embryo implantation

## Abstract

Early pregnancy failure occurs when a mature embryo attaches to an unreceptive endometrium. During the formation of a receptive endometrium, extracellular vesicles (EVs) of the uterine fluids (UFs) deliver regulatory molecules such as small RNAs to mediate intrauterine communication between the embryo and the endometrium. However, profiling of small RNAs in goat UFs’ EVs during pregnancy recognition (day 16) has not been carried out. In this study, EVs were isolated from UFs on day 16 of the estrous cycle or gestation. They were isolated by Optiprep™ Density G radient (ODG) and verified by transmission electron microscopy (TEM), nanoparticle tracking analysis (NTA), and Western blotting. Immunostaining demonstrated that CD63 was present both in the endometrial epithelium and glandular epithelium, and stain intensity was greater in the pregnant endometrium compared to the non-pregnant endometrium. Small RNA sequencing revealed that UFs’ EVs contained numerous sRNA families and a total of 106 differentially expressed miRNAs (DEMs). Additionally, 1867 target genes of the DEMs were obtained, and miRNA–mRNA interaction networks were constructed. GO and KEGG analysis showed that miRNAs were significantly associated with the formation of a receptive endometrium and embryo implantation. In addition, the fluorescence in situ hybridization assay (FISH) showed that chi-miR-451-5p was mainly expressed in stromal cells of the endometrium and a higher level was detected in the endometrial luminal epithelium in pregnant states. Moreover, the dual-luciferase reporter assay showed that chi-miR-451-5p directly binds to PSMB8 and may play an important role in the formation of a receptive endometrium and embryo implantation. In conclusion, these results reveal that UFs’ EVs contain various small RNAs that may be vital in the formation of a receptive endometrium and embryo implantation.

## 1. Introduction

Goats are valuable livestock breeds as they provide fiber, meat, milk, and other by-products [1], and have promising importance in biomedicine and the transgenic production of pharmaceutical drugs such as human enzymes [2]. Reproduction has a critical impact on goat production, but early pregnancy failure is an important factor limiting the reproductive efficiency of goat. Early pregnancy failure occurs when a mature embryo attaches to a non-receptive endometrium, hindering reproduction and herd development [3]. The endometrium undergoes remarkable changes during the formation of a receptive state, including the proliferation of endometrial stromal cells (ESCs), differentiation of endometrium epithelial cells (EECs), and the acquisition of adhesion properties that allow the embryo to attach and subsequently invade [4,5,6]. Numerous studies have shown that a non-receptive endometrium can lead to transplantation failure of the in vitro fertilization embryo [7,8]. Furthermore, it has been suggested that uterine fluids’ (UFs) extracellular vesicles (EVs) can affect endometrial receptivity and implantation both in human [9] and ovine pregnancies [10]. Therefore, further research is needed to explore the specific regulatory mechanisms of UFs’ EVs underlying the endometrial changes during the formation of a receptive state to improve animal husbandry development and embryo transplantation success rate.

EVs, including exosomes (30–150 nm) and microvesicles (100–1000 nm), are a heterogeneous group of cell-derived membranous structures [11], which carry and transport regulatory molecules, such as microRNAs (miRNAs), mRNAs [12], lipids [13] and proteins [14], mediating intercellular communication in cells and tissues [15]. Extracellular vesicles have been isolated and characterized in UFs in humans [16], ovine [10], mice [17], bovine [18] and porcine [19]. During the peri-implantation period, embryonic cells uptake endometrium-derived EVs. Similarly, embryo-derived EVs modulate uterine function [20,21]. In order to better characterize endometrial receptivity, recent research focused on the optimization of endometrial biopsies using UFs’ EVs [22], the exploration of UFs’ EVs transcriptomic changes during endometrial window [23], and the identification of UFs’ EVs small non-coding RNA biomarkers for endometrial receptivity and implantation success [24].

MiRNAs are small non-coding RNAs consisting of 19–25 nucleotides that participate in many biological processes, including apoptosis, cell differentiation, cell proliferation, and tumorigenesis, by regulating the post-transcriptional silencing of target genes [25,26]. MiRNAs such as miR-30d [16] amd miR-183-5p [27] play an important role in endometrial receptivity in humans. Additionally, the function of miR-183 [28], miR-23a-3p [29] and miR-30a-3p [30] in endometrial receptivity has also been detected in mice. However, additional research is needed to clarify the biological function of these miRNAs in endometrial receptivity in goats.

In this study, EVs were obtained, isolated, and characterized from goat UFs of both the estrous cycle and gestation on day 16 (C16 and P16), as embryo implantation begins around day 15 to 16 of the pregnancy [31]. The expression levels of CD63 protein on the endometrium were detected using immunohistochemistry. The expression of small RNAs in these extracellular vesicles was then profiled by small RNA sequencing. Moreover, the FISH assay was used in the detection of chi-miR-451-5p in the endometrium in non-pregnant and pregnant states, while the dual-luciferase reporter assay was used to verify the accuracy of the predicted target gene.

The present findings add to the current understanding of the biological function of miRNAs in endometrial receptivity. This study provides ideas for improving the efficiency of embryo transplantation, and treatment of infertility therapy.

## 2. Materials and Methods

### 2.1. Sample Collection

Six healthy, primiparous Chuanzhong black goats (*Capra hircus*) were obtained from Guangdong Wen’s Foodstuffs Group Co., Ltd. (Yunfu, China). The animals were randomly assigned to the cyclic (C16, *n* = 3) and pregnant (P16, *n* = 3) groups. Goats that belonged to the pregnant group were artificially inseminated using extended semen from one ram at the onset of estrus (day 0) and again 12 h after. Uteri were obtained from goats slaughtered on day 16, and UFs was collected after uteri removal by flushing with 30 mL of Dulbecco’s phosphate-buffered saline (DPBS; GIBCO, Cat. No. 14190144). Pregnancy was confirmed by the presence of apparently normal filamentous conceptuses in uterine flushing [32].

### 2.2. UFs’ EVs Isolation

EVs were isolated from UFs by modification of the Optiprep™ Density Gradient (ODG) as previously described [33]. Briefly, the UFs were centrifuged at 2000× *g* for 30 min at 4 °C to remove impurities, followed by ultra-centrifugation at 130,000× *g* using an Optima XPN-100 Ultracentrifuge (Beckman Coulter) with an SW 32 Ti rotor (k-Factor 204, Beckman Coulter) for 1 h at 4 °C to obtain the EVs. The EV pellets were re-suspended in 500 µL of PBS and loaded to the top of a discontinuous OptiPrep™ density gradient, layered as 3 mL of 40% solution, 3 mL of 20% solution, 3 mL of 10% solution, and 2.5 mL of 5% solution. The solution was prepared by diluting iodixanol in PBS (60% *w/v*; Sigma Aldrich, Cat. No. D1556). PBS was then added to the gradient to achieve the required tube fill volume. The tubes were then ultra-centrifuged at 100,000× *g* using an Optima XPN-100 Ultracentrifuge (Beckman Coulter) with an SW 32 Ti rotor (k-Factor 204, Beckman Coulter) for 16 h. From the top to the bottom, PBS was collected as fraction 0 and the OptiPrep™ density gradient volume was collected as independent fractions of 1 mL each, which were then washed in PBS for a further 90 min and ultra-centrifuged at 100,000× *g* using an Optima XPN-100 Ultracentrifuge (Beckman Coulter) with an SW 32 Ti rotor (k-Factor 204, Beckman Coulter). Finally, the supernatant was removed, and the pellet was re-suspended in PBS for further analysis.

### 2.3. Transmission Electron Microscopy

The morphology of EVs from UFs was determined using transmission electron microscopy (TEM; Talos L120C, Thermo Fisher) based on a previously described method [34,35]. In brief, the re-suspended EVs were loaded onto Cu grids and incubated at room temperature for 10 min. Then, the grids were stained with 2% uranyl acetate for 2 min and dried overnight at room temperature.

TEM analysis was also used to assess the presence of EVs in the endometrium by modification of a previously described method [36]. Briefly, tissues were dehydrated in alcohol, embedded in epoxy resin, ultra-sectioned (less than 1 mm^3^), and transferred to 300-mesh Formvar coated nickel grids (Electron Microscopy Sciences). A mixture of 4% uranyl acetate and lead citrate was then added to the sections for 15 min, and the grids were dried overnight at room temperature. All 6 tissue samples (C16, *n* = 3; C16, *n* = 3) were analyzed for TEM. The grids were then observed under a transmission electron microscope and the images were analyzed.

### 2.4. Nanoparticle Tracking Analysis

The size distribution of EVs were analyzed by nanoparticle tracking analysis (NTA). EV samples of 1 µL were diluted with PBS at the ratio of 1:50 before NTA, and Zeta View (MX110, Particle Metrix) with a 488 nm laser wavelength was used to assess the size distribution. The pre-acquisition parameters were: a sensitivity of 90, a shutter speed and laser pulse duration of 70, a temperature of 26.8 °C, and pH of 7.0. Post-acquisition parameters were set to a minimum brightness of 30, a maximum area of 1000 pixels, and a minimum area of 10 pixels. Additionally, more than 1000 particles were analyzed for size determination.

### 2.5. Western Blot Analysis

The proteins from UFs‘ EVs and the endometrium were extracted by RIPA buffer (Beyotime, Cat. No. P0013B) supplemented with 1% PMSF (Beyotime, Cat. No. ST506). The sample was denatured by heating, separated by SDS-PAGE, transferred to a polyvinylidene difluoride (PVDF) membrane (Millipore, Cat. No. IPVH08110), washed four times (5 min each time) with TBST, and blocked with 5% skim milk in TBST for 2 h at room temperature. The following antibodies were incubated with the membranes after being washed four times with TBST: rabbit anti TSG101 (ZEN-BIOSCIENCE, Cat. No. abs127362, 1:1000 in TBST), rabbit anti HSP70 (Proteintech, Cat. No. 25682-1-AP, 1:1000 in TBST), and rabbit anti Calnexin (Proteintech, Cat. No. 10427-2-AP, 1:1000 in TBST) overnight at 4 °C. After being washed four times with TBST, the membranes were incubated with HRP-labeled Goat Anti-Rabbit secondary antibodies (Beyotime, Cat. No. A0208, 1:1000 in TBST) for 2 h at room temperature. The images of the membranes were captured by a UVP system (Upland) after they were treated with an enhanced chemiluminescence (ECL, Beyotime, Cat. No. P0018S) reagent.

### 2.6. Immunohistochemistry

Immunohistochemical analysis was performed as described previously [37]. First, sections (4 μm thick) of endometrium were deparaffinized with xylene and sequentially rehydrated with 100% ethanol, 95% ethanol, 90% ethanol, 80% ethanol, 70% ethanol, and distilled water. The sections were then blocked with 3% hydrogen peroxide (H_2_O_2_) for 15 min at room temperature, rinsed with distilled water, and treated thrice with 0.01 M sodium citrate buffer (pH 6.0) in a microwave oven at 750 W (5 min each time). The sections were then allowed to cool at room temperature for 30 min, washed with PBS three times, and blocked with 5% bovine serum albumin (BSA, Sigma, Cat. No. A1933) in PBS for 30 min. Rabbit anti CD63 (Abcam, Cat. No. ab216130, 1:50 in PBS) was used to incubate the sections at 4 °C overnight in a humid chamber. After rinsing with PBS three times, the sections were incubated with Biotin-labeled Goat Anti-Rabbit IgG (Beyotime, Cat. No. A0277, 1:100 in PBS) at room temperature for 40 min. The sections were then washed with PBS three times, visualized with DAB (Beyotime, Cat. No. P0203), counterstained with hematoxylin, and mounted. For each sample, the corresponding non-specific IgG was used to replace the primary antibody as a negative control.

An Olympus microscope BX-53 (Olympus) with a digital camera DP26 and Adobe Photoshop CS6 (Adobe Systems Inc.) was used to record and assemble the images. ImagePro Plus 6.0 software (Media Cybernetics, Silver Spring) was used to calculate the mean integrated optical density (IDO) by quantifying the immunohistochemical staining intensity as previously described [38].

### 2.7. RNA Extraction and sRNA Sequencing

The RNAs of UFs’ EVs was isolated by the exoRNeasy Serum/Plasma Maxi Kit (Qiagen, Cat. No. 77023) according to the manufacturer’s instructions. RNA purity and integrity was determined using the NanoPhotometer^®^ spectrophotometer (IMPLEN, CA, USA) and Agilent 2100 pic600 (Agilent Technologies). Approximately 20 ng of total RNA from three cyclic and three pregnant UFs’ EVs were used as input material for the small RNA library construction according to the manufacturer’s instructions for the NEBNext^®^ Multiplex Small RNA Library Prep Set for Illumina^®^ (NEB). Sequencing was carried out on an Illumina Hiseq 2500 platform (Illumina, SanDiego, CA, USA) at the Novogene (Beijing, China).

### 2.8. sRNA Sequence Data Analysis

First, clean reads were processed from raw data using customized Perl and Python scripts. The following reads were removed: reads with an *n* proportion greater than 10%, reads with 5′ adapter contaminants, reads lacking a 3′ adapter or the insert tag, reads containing poly A, T, G, or C, and low-quality reads. The clean reads were mapped to the *Capra hircus* reference sequence by Bowtie (v.0.12.9) [39]. Subsequently, RepeatMasker (v.4.0.3) and Rfam database (ftp://selab.janelia.org/pub/Rfam/, accessed on 30 July 2021) [40] were used to map clean reads to mRNA, rRNA, tRNA, snRNA, snoRNA, and repeat gene sequences. Next, mirDeep2 (v.2.0.0.5) [41] and srna-tools-cli (http://srna-tools.cmp.uea.ac.uk/, accessed on 30 July 2021) were used to predict potential miRNAs, while miREvo [42] and mirDeep2 were used to identify novel miRNAs.

### 2.9. Differentially Expressed (DE) Small RNAs

The expression levels of miRNAs were calculated using Transcript Per Kilobase Million (TPM) as described in a previous report [43]. Thereafter, DESeq2 [44] was used to determine the differential expression, while the Benjamini–Hochberg method [45] was used to adjust the raw *p*-value to the corrected *p*-value, and miRNAs with read numbers |foldchange| ≥2 and *p*-value ≤ 0.05 were considered as a DE miRNA.

### 2.10. Real Time Quantitative PCR (RT-qPCR)

The RNA samples from three cyclic and three pregnant UFs’ EVs were analyzed by quantitative PCR (RT-qPCR). Six differentially expressed miRNAs were randomly selected (chi-miR-140-5p, chi-miR-10b-5p, chi-miR-127-3p, chi-miR-17-5p, chi-miR-200a, chi-miR-30a-5p). Reverse transcription was performed with the Mir-X miRNA First-Strand Synthesis Kit (Takara, Cat. No. 638315), according to the manufacturer’s protocols. Subsequently, these cDNAs were validated by qPCR using PowerUp™ SYBR™ Green Master Mix (Thermo Fisher, Cat. No. A25742) on a Real-Time PCR System (Applied Biosystems). The spike-in control cel-miR 39-1 (Qiagen, Cat. No. 219610) was used as the endogenous control. The forward primer sequences are visible in Supplementary Table S2, while the reverse primer was obtained from the above kit. The efficiencies of the amplification curves and the quantification cycle (Cq) were determined using LinRegPCR software [46], and then the relative expression levels of miRNA were calculated using the 2^−△△^Ct method [47].

### 2.11. Prediction of miRNA Target Genes

Three algorithm tools, miRanda [48], targetscan [49], and RNAhybrid [50], were used to predict the target genes. The intersected prediction of the three tools was selected for further analysis. Subsequently, Cytoscape (v.3.7.2, http://www.cytoscape.org/, accessed on 30 July 2021) [51] was used to analyze the miRNA-gene regulatory network between differentially expressed miRNAs (DEMs) and target genes.

### 2.12. Functional Annotation of the Predicted Target Genes

GOseq R package [52] was used to analyze the Gene Ontology (GO) enrichment analysis of predicted target genes. GO terms with a corrected *p*-value < 0.05, adjusted by the Benjamini–Hochberg method, were considered significantly enriched. The main biochemical metabolic pathway and signal transduction pathway of predicted target genes were analyzed based on the Kyoto Encyclopedia of Genes and Genomes (KEGG) [53] using KOBAS software [54].

### 2.13. Fluorescence In Situ Hybridization

FISH assays were performed to detect chi-miR-451-5p in endometrium tissue as previously described [55]. Cy3-labeled chi-miR-451-5p probes were designed and synthesized by Servicebio. The section stained with hybridization buffer without a probe was used as a negative control. Nuclei were stained with DAPI. All 6 tissue samples (C16, *n* = 3; C16, *n* = 3) were analyzed with FISH. Images were taken by microscopy (Nikon Eclipse ci) with an imaging system (Nikon DS-U3).

### 2.14. Dual-Luciferase Reporter Assay

Normal and mutant 3′UTRs (untranslated regions) of the *PSMB8* gene (Gene ID: 102180902) in goats were amplified by PCR and inserted downstream of the luciferase gene in the pGL3 vector (Promega, Cat. No. E1751). The sequence of *PSMB8* 3′UTR and *PSMB8*-mut 3′UTR were as follows: 5′-CAATAAAGGAAAACGGTTA-3′ and 5′-CAATAAAGGAAGGTAACCA-3′. 293T cells used for the dual-luciferase reporter assay were purchased from CCTCC (Wuhan Province, China). Co-transfection with chi-miR-451-5p mimics (or chi-miR-451-5p mimics NC) and *PSMB8*-3′UTR (or *PSMB8*-Mut or pGL3 vector) was performed using Lipofectamine 3000 (Invitrogen, Cat. No. L3000015), together with 0.1 µg/well of pRL-TK (Beyotime, Cat. No. D2760) when the 293T cells reached a confluence of 75%. Forty-eight hours following the transfection, firefly luciferase activities and Renilla luciferase activities were measured continuously using a dual-luciferase reporter assay system (Beyotime, Cat. No. RG027) according to the manufacturer’s instructions on a modular multimode microplate reader (BioTek Synergy H1) at 560 nm and 465 nm, respectively. The firefly to Renilla luciferase ratio was used to measure relative activity.

### 2.15. Statistical Analysis

Prism 8.0 (GraphPad) was used to perform data analysis and a two-tailed Student’s t-test was used to compare two groups. The normal distribution assumption was tested using the Shapiro–Wilk (W) test and the Kolmogorov–Smirnov (distance) test, and the equal variances were assessed by the F-test before using the Student’s t-test. * *p* < 0.05 was considered statistically significant; ** *p* < 0.01 was considered especially significant.

## 3. Results

### 3.1. Characterization of UFs’ EVs

**Figure 1:**
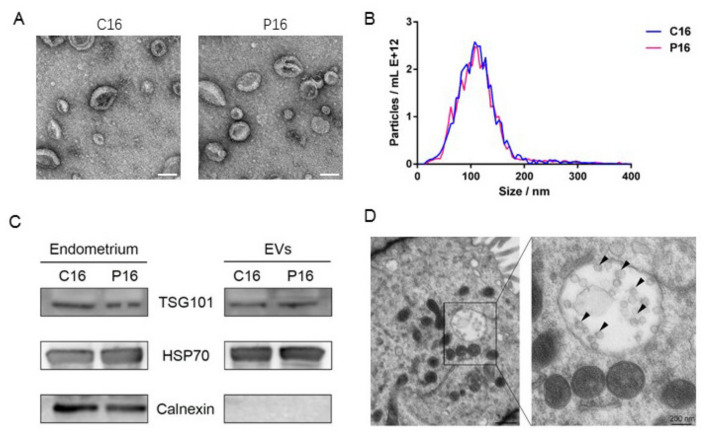
Characterization of EVs from porcine UFs. (**A**) TEM analysis of extracellular vesicles from goat UFs. Scale bar = 100 nm. (**B**) NTA of the extracellular vesicles from goat UFs. (**C**) Western blotting detected EV protein markers, TSG101 and HSP70, in the UFs’ EVs fraction and the endometrium. Endoplasmic reticulum membrane marker (Calnexin) was only detected in the endometrium. (**D**) TEM analysis of endometrial luminal epithelium on day 16 of pregnancy (*n* = 3). Legend: C16, on day 16 of the estrous cycle; P16, on day 16 of pregnancy. Arrowhead refers to EVs.

**Figure 2:**
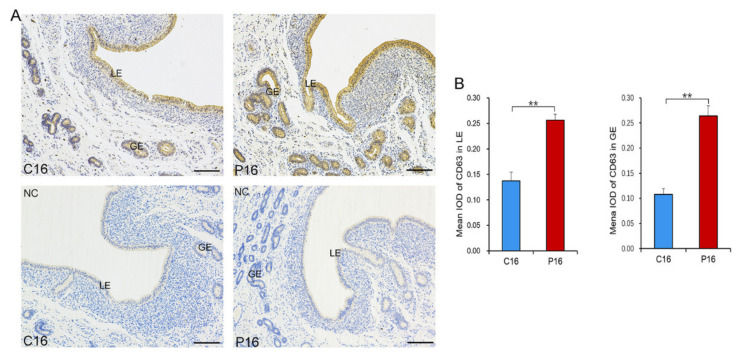
CD63 levels in pregnant and non-pregnant endometrium. (**A**) Expression pattern of CD63 in pregnant and non-pregnant endometrium. Scale bar = 200 μm. (**B**) Quantitative analysis of CD63 by measuring the average integrated optical density (IOD) in LE and GE of pregnant and non-pregnant endometrium. Data are shown as the mean ± SEM values (*n* = 3). Legend: C16, on day 16 of the estrous cycle; P16, on day 16 of pregnancy; LE, endometrial luminal epithelium; GE, glandular epithelium. ** *p* < 0.01 was considered especially significant.

The morphology of UFs’ EVs in goats was evaluated by TEM. UFs’ EVs appeared as cup-shaped vesicular structures (Figure 1A) that were mainly detected in ODG fractions 9, 10, and 11. NTA analysis showed that the UFs’ EVs had an average diameter of between 30 and 200 nm (Figure 1B). Western blotting showed that UFs’ EVs were positive for specific EV protein markers (TSG101 and HSP70) and negative for endoplasmic reticulum membrane marker (calnexin) (Figure 1C). We concluded that the isolated UFs’ EVs had all the characteristics of EVs. TEM analysis was performed to determine the origin of EVs in UFs and EVs were detected in the endometrial luminal epithelium (Figure 1D). Immunohistochemistry was used to confirm the different levels of CD63 between C16 and P16. This analysis detected higher levels of CD63 in the endometrial luminal epithelium (LE) and glandular epithelium (GE) of the pregnant endometrium (Figure 2).

### 3.2. Overview of the Sequencing Data

In this study, we collected six UFs’ EVs from six goats on day 16 of the estrous cycle and gestation. Illumina Hiseq 2500 platform was used to detect the expression profiles of sRNAs in UFs’ EVs samples. C16 and P16 obtained a total of 16,383,161 and 15,358,745 paired-end raw reads, respectively (Table 1). After sequencing and data filtration, a total of 15,691,455 and 13,332,890 clean reads were obtained, respectively. To identify potential novel miRNAs in UFs’ EVs, RepeatMasker and the Rfam database were used, and 12,387,113 and 8,560,201 sRNA sequences were obtained in C16 and P16, respectively. About 2.45% (297,961/12,387,113) and 4.97% (387,875/8,560,201) of sRNA sequences were identified as the known miRNAs, while 0.005% (558/12,387,113) and 0.05% (3519/8,560,201) were identified as the novel miRNAs. The remaining sequences formed other types of RNA, including exon, intron, repeats, rRNA, snoRNA, snRNA, and tRNA. In addition, the Pearson correlation coefficient was used to check the reliability of the biological duplication and data quality (Figure S1).

### 3.3. Differentially Expressed miRNAs in Goat UFs’ Evs

**Figure 3:**
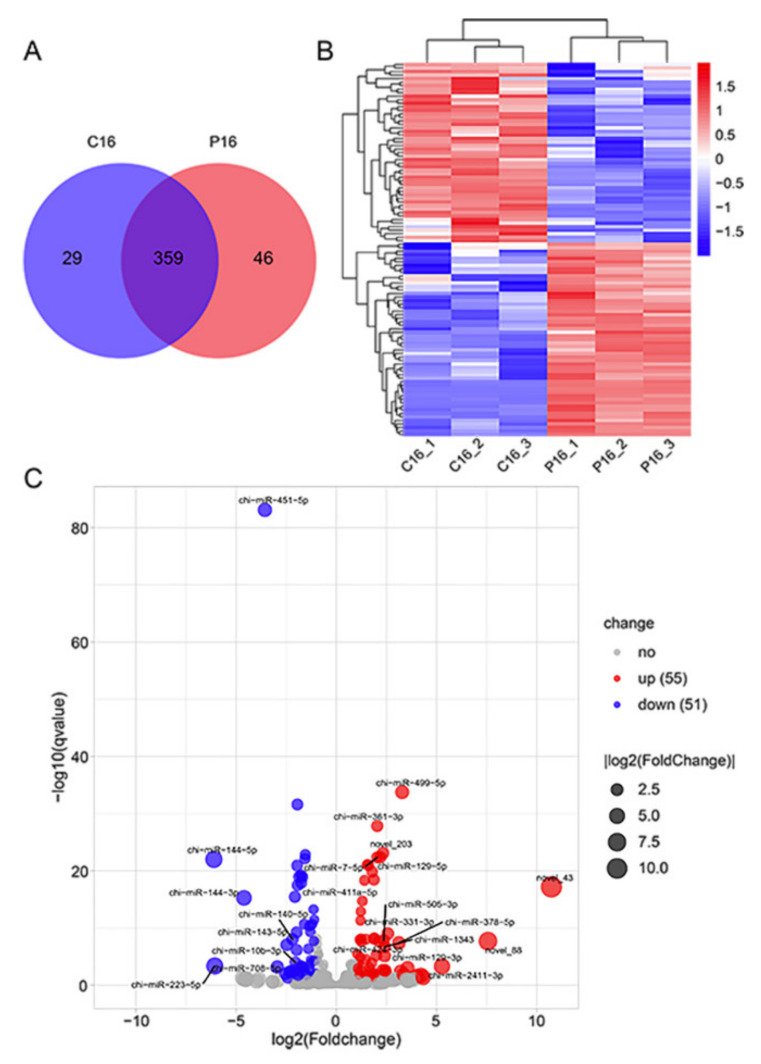
Overall differences of miRNAs in pregnant and non-pregnant state. (**A**) The Venn diagram shows the differential expression of miRNAs. (**B**) Heatmap shows the expression patterns of miRNAs between C16 and P16. (**C**) The volcano plots of the differentially expressed miRNAs on P16 and C16. Legend: C16, on day 16 of the estrous cycle; P16, on day 16 of pregnancy.

**Figure 4:**
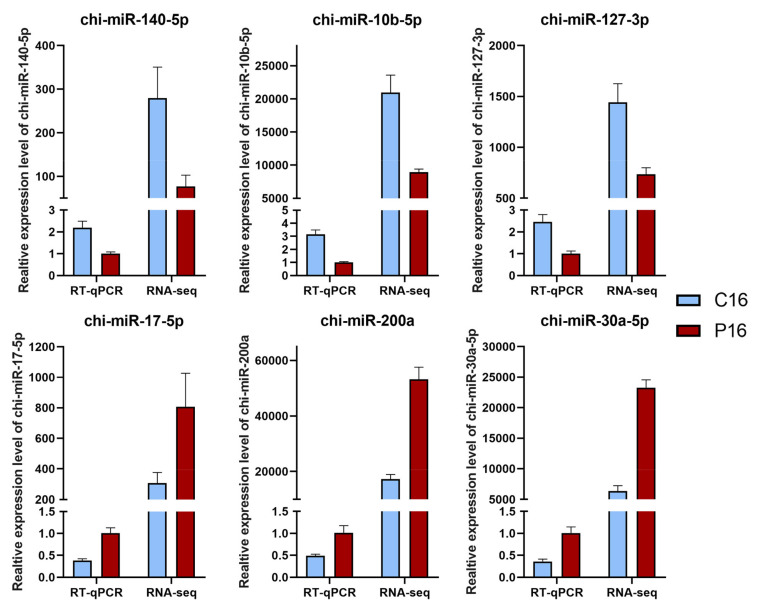
Validation of the expression level of miRNAs. The randomly selected DEMs were verified by RT-qPCR and the results showed was consistent with the RNA-seq data. Data are presented as mean ± SEM values (*n* = 3).

A comparison of miRNAs expression levels in goat Ufs’ Evs between C16 and P16 showed that 359 miRNAs were co-expressed, while 29 and 46 miRNAs were specifically expressed in C16 and P16, respectively (Figure 3A). There were 106 DEMs with a |foldchange| ≥ 2 and *p*-value ≤ 0.05 (Table S1). Among the miRNAs, 55 were upregulated, while 51 were downregulated in P16 compared to C16 (Figure 3C and Table S1). The expression patterns of DEMs in non-pregnant and pregnant states were analyzed by hierarchical clustering. This revealed that miRNAs were similarly expressed in the same state but differentially expressed between pregnant and non-pregnant states (Figure 3B). A random selection of six DEMs (chi-miR-140-5p, chi-miR-10b-5p, chi-miR-127-3p, chi-miR-17-5p, chi-miR-200a, chi-miR-30a-5p) for the validation of miRNA expression levels using RT-qPCR showed consistent results with RNA sequencing (Figure 4).

### 3.4. Target Gene Predictions for DEMs

**Figure 5:**
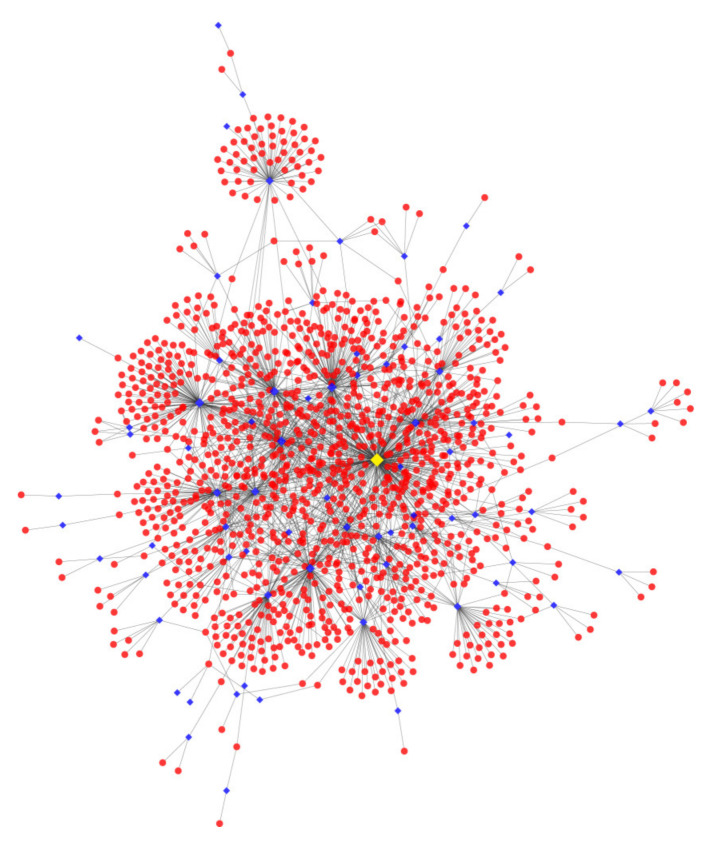
DEMs’ target genes regulatory network. The red circle represents target genes, the rhombus represents DEMs and the yellow rhombus represents chi-miR-1343.

MiRNAs mediate posttranscriptional gene silencing of the target genes by targeting the 3′ UTR of mRNA. The seed region in nucleotides 2 to 7 at the end of miRNA is a crucial sequence [26]. To further investigate the effects of miRNAs from C16 and P16 UFs’ Evs during the peri-implantation period, three software systems (miRanda, targetscan, and RNAhybrid) were used to predict the target genes. Consequently, 1867 target genes from DEMs were identified (Table S3). Target gene regulatory networks showed that chi-miR-1343 targets 284 mRNAs, which is highest in DEMs, and that an mRNA can also be regulated by multiple miRNAs (Figure 5).

### 3.5. Go and KEGG Pathway Analyses of the Predicted Target Genes

**Figure 6:**
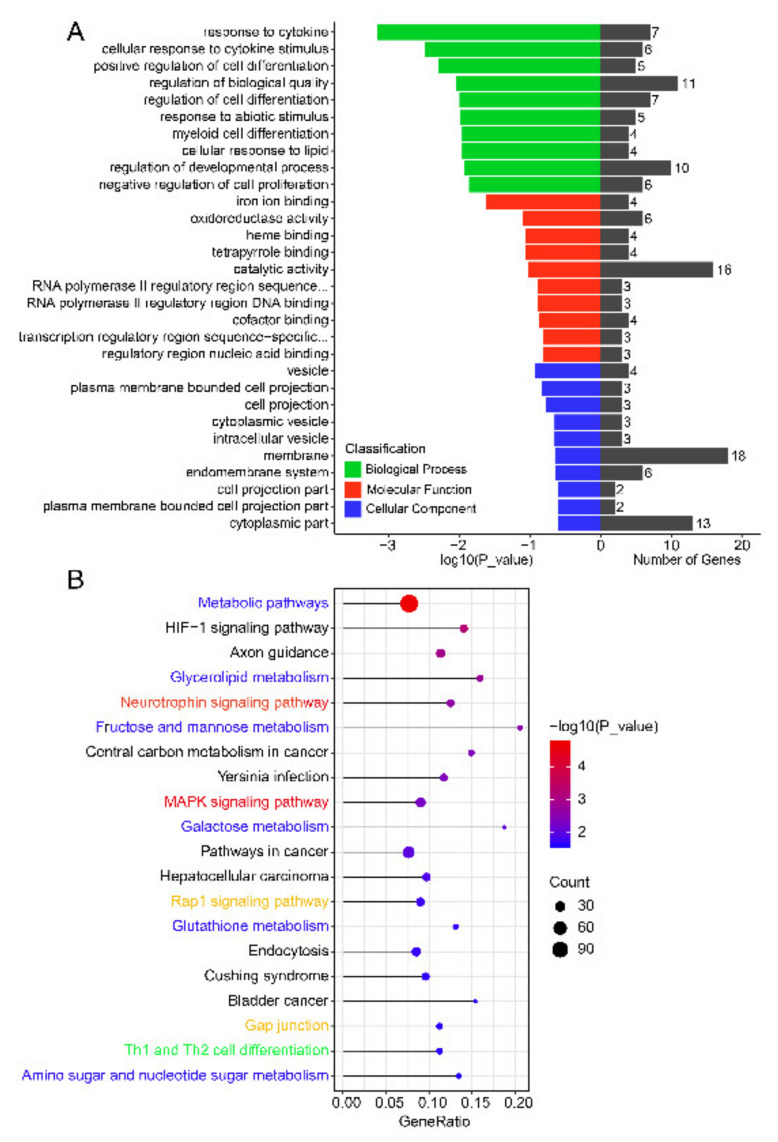
GO and KEGG enrichment analysis of predicted target genes of DEMs. (**A**) GO analysis of predicted target genes of SEMs. (**B**) Top 20 KEGG pathways of predicted target genes of DEMs. The x-axis indicates the ratio of the predicted target genes to the annotated genes enriched in this pathway, while the y-axis indicates the KEGG pathway. “Count” means the number of predicted target genes enriched in this pathway. The color represents the degree of enrichment, with red representing significant enrichment. The red font indicates a relationship between the pathways and endometrial development and remodeling. The yellow font indicates a relationship between the pathways and embryo implantation. The blue font indicates a relationship between the pathways and metabolism. The green font indicates a relationship between the pathways and maintenance of pregnancy.

GO analysis revealed that the target genes were mainly enriched during immune processes, cell differentiation, cell development, and cell proliferation. These processes are related to endometrial receptivity and embryo implantation, which involves the response to cytokine, the cellular response to cytokine stimulus, the positive regulation of cell differentiation, the regulation of cell differentiation, the response to abiotic stimulus, myeloid cell differentiation, the cellular response to lipids, the regulation of developmental process, and the negative regulation of cell proliferation (Figure 6A and Table S4). KEGG pathway analysis showed that some pathways, such as the Neurotrophin signaling pathway and the MAPK signaling pathway, can be related to endometrial development and remodeling due to their function in cell development and differentiation [56,57]. In addition, some pathways, such as the Rap1 signaling pathway and Gap junction, were related to embryo implantation, because Rap1 can induce cell–cell junction stabilization and endothelial cell sprouting [58]. Moreover, some pathways were related to metabolisms, such as metabolic pathways, glycerolipid metabolism, fructose and mannose metabolism, galactose metabolism, glutathione metabolism, and amino sugar and nucleotide sugar metabolism. Besides, Th1 and Th2 cell differentiation was related to the maintenance of pregnancy because of the prerequisite of balanced Th1/Th2 cytokines in successful pregnancy [59] (Figure 6B and Table S5).

### 3.6. Chi-miR-451-5p Expressed in Stroma Cell of the Endometrium

**Figure 7:**
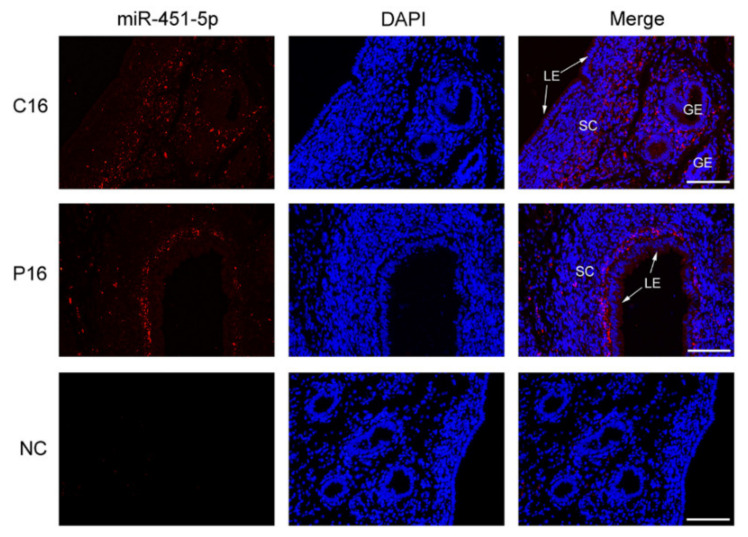
Chi-miR-451-5p expressed in stromal cells of the endometrium. Fluorescence in situ hybridization (FISH) assays for miR-451-5p in the endometrium on C16 and P16 tissue samples (*n*=3). Scale bar = 100 μm. Legend: C16, on day 16 of the estrous cycle; P16, on day 16 of pregnancy; LE, endometrial luminal epithelium; GE, glandular epithelium; SC, stroma cell.

A comparison of the non-pregnant and pregnant goat UFs’ EVs on day 16 revealed that chi-miR-451-5p was significantly down-regulated with the highest *p*-value. FISH assays were conducted to further validate miRNA expression levels and determine the location of miR-451-5p in the endometrium on C16 and P16. Chi-miR-451-5p was mainly expressed in the stroma cells of the endometrium, showing that stroma cells may be involved in the secretion of chi-miR-451-5p. Additionally, a higher expression level of chi-miR-451-5p was detected in the endometrial luminal epithelium on P16 (Figure 7).

### 3.7. PSMB8 Is the Direct Target Gene of miR-451-5p

**Figure 8:**
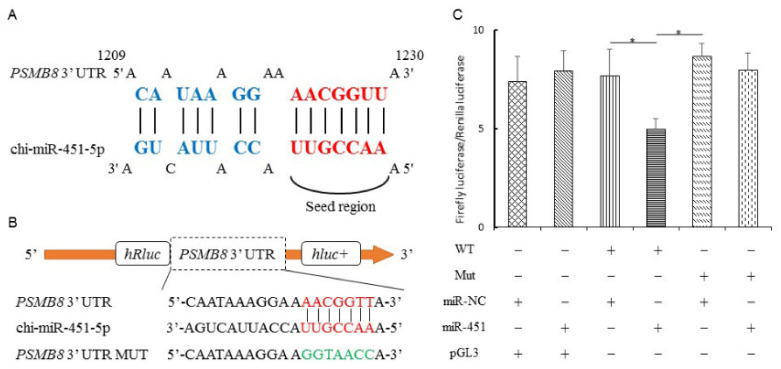
Chi-miR-451-5p targets the 3′ UTR of PMSB8. (**A**) The predicted binding site of chi-miR-451-5p in the 3′UTR of PSMB8. (**B**) The design of luciferase reporter. PSMB8 3′ UTR sequence contains the chi-miR-451-5p binding site; PSMB8 3′ UTR Mut sequence has mutations of the chi-miR-451-5p binding site. (**C**) 293T cells co-transfected with luciferase reports of PMSB83′ UTR (WT) or PMSB83′ UTR (Mut) and chi-miR-451-5p mimics or negative control (NC) before the luciferase reporter assay. Data are shown as the mean ± SEM values (*n* = 3). * *p* < 0.05 was considered statistically significant.

The target gene of miR-451-5p, *PSMB8,* is known as a direct regulator of cell migration, proliferation, and the apoptosis of glioma cells through modulating ERK1/2 and PI3K/AKT signaling pathways [60]. This may play an important role in endometrial remodeling, as embryo implantation has common pathways with tumor metastasis during invasion and angiogenesis [61]. Thus, a dual-luciferase reporter assay was used to verify the predicted target gene. This revealed significantly suppressed luciferase activity in the wild-type miRNA mimic. However, the luciferase activity of the mutant group was unchanged, indicating that chi-miR-451-5p specifically inhibits the *PSMB8* gene (Figure 8).

## 4. Discussion

EVs have been reported to mediate cell–cell communication and modulate numerous biological processes, including endometrial receptivity, which is essential in embryo implantation [62]. Here, we isolated goat UFs’ EVs and validated them through TEM, NTA, and Western blotting. The UFs’ EVs showed typical EV features, such as cup-shaped morphology with a diameter of 30–200 nm, EV protein markers (TSG101 and HSP70) and lack of endoplasmic reticulum membrane marker (calnexin), verifying the purity of the EVs. Endometrium expressing EV markers (TSG101 and HSP70) and EVs were seen in the endometrial luminal epithelium using TEM, suggesting that the endometrial luminal epithelium could secrete EVs. In addition, immunohistochemistry showed LE and GE in P16 had higher CD63 levels, which was consistent with previous studies that CD63 stain increased from the proliferative to secretory phase reach a maximum at the time of endometrial receptivity [9]. However, embryos can also secrete EVs, and these EVs can be taken up by the maternal side [63]. Therefore, a further study is required to investigate the origin of UFs’ EVs.

During embryo implantation, endometrium secretes integrins and adhesion molecules that regulate cell adhesion and motility [64] as well as several endometrial molecules such as miRNAs [65]. MiRNAs mediate embryo implantation by post-transcriptional regulation. Studies have shown that miRNA is related to endometrial receptivity [5,66] and embryo implantation [67,68]. In the present study, small RNA sequencing was used to compare UFs’ EVs small RNAs expression levels in non-pregnant and pregnant states. A total of 297961 known miRNAs and 558 novel miRNAs were detected in the non-pregnant state, while 387875 known miRNAs and 3519 novel miRNAs were detected in the pregnant state. A total of 106 of these miRNAs were differentially expressed between non-pregnant state and pregnant state.

Among these miRNAs, novel-43, novel-88, chi-miR-2411-3p, novel-36 were significantly increased in the UFs’ EVs in pregnancy compared to the non-pregnancy state (the top four most significantly up-regulated miRNAs). However, chi-miR-144-5p, chi-miR-223-5p, chi-miR-144-3p, and chi-miR-451-5p showed a significant decrease in expression (the top four most significantly down-regulated miRNAs). Studies have shown that miR-451-5p participates in embryo implantation by targeting Ankrd46 [69] and can influence the embryonic potential of mice and humans [70]. Moreover, miR-451-5p may regulate cell proliferation and invasion by targeting mRNAs that are members of signaling pathways, such as P13 K/AKT [71,72], NOTCH1 [73], STAT3/HIF [74], and p38/MAPK [75], which are well-known pathways in embryo implantation [69]. In this study, the FISH assay was used to further verify the differential expression of Chi-miR-451-5p in the endometrium between C16 and P16. Results showed that Chi-miR-451-5p was mainly expressed in the stromal cells of the endometrium, suggesting that stromal cells may play a role in the secretion of chi-miR-451-5p. Additionally, higher chi-miR-451-5p levels were detected in the endometrial luminal epithelium on P16, as communication between the endometrial luminal epithelium and embryo is important for embryo implantation [76]. Moreover, the dual-luciferase reporter assay proved that chi-miR-451-5p directly targets 3′-UTR of *PSMB8*. Previous research has shown that *PSMB8* regulates glioma cell differentiation [77], cell migration, proliferation, and apoptosis [60], which are similar processes to embryo implantation [61]. This suggests that *PSMB8* is potentially essential in the formation of a receptive endometrium and embryo implantation. Numerous DEMs revealed by sequencing, such as miR-143-5p [78], miR-126-3p [79], miR-141 [80], miR-199a-5p [81], miR-34a [82], and miR-200a [83], have also been associated with endometrial receptivity and embryo implantation.

GO analysis showed that the top 10 biological processes were mainly enriched in immune processes, cell differentiation, cell development, and cell proliferation. These processes are related to endometrial receptivity and embryo implantation. KEGG pathway analysis showed that the target genes were associated with endometrial development and remodeling, embryo implantation, metabolism, and maintaining pregnancy. Among the top 20 pathways, the “Neurotrophin signaling pathway” and “MAPK signaling pathway” attracted our attention. Neurotrophins are a family of trophic factors involved in the differentiation and survival of neural cells [84], which showed that these genes may be related to the development of the nervous system during the formation of a receptive endometrium. In addition, Neurotrophins exert their functions by interacting with the tyrosine kinase receptors (Trks) to activate different pathways [85]. Neurotrophins can activate Ras, which is essential in the differentiation of neural cells promoting neural subpopulations survival, and activate downstream signaling of the MAPK pathway [86]. The MAPK signaling pathway has been associated with cell proliferation, differentiation, migration, senescence, and apoptosis [57], which can contribute to endometrium receptivity. Moreover, the “Rap1 signaling pathway”, which regulates cell adhesion [87] and cell junction [58], was enriched in the KEGG pathway analysis. Some pathways are related to cancers, such as bladder cancer, as embryo implantation shares common pathways with tumor metastasis during invasion and angiogenesis [61]. Therefore, an in-depth study on embryo implantation mechanisms may offer a breakthrough in the treatment of malignant tumors.

Collectively, these results suggest that DEMs in goat UFs’ EVs have potentially vital effects on endometrial receptivity and embryo implantation, but a verification in larger cohorts both with an intensive study on the in vitro regulation functions of UFs’ EVs miRNAs are required. Besides, both endometrium and embryonic cells can secrete and uptake EVs [20], but the source of UFs’ EVs warrants further study. In addition, as EVs miRNAs also carry and transport mRNAs, proteins, and other regulatory molecules during embryo implantation, a comprehensive sequencing analysis to reveal the regulatory mechanism of goat UFs’ EVs, such as piRNA that promotes fertility, is necessary.

## 5. Conclusions

In conclusion, we isolated and characterized goat UFs’ EVs in pregnant and non-pregnant endometrium. Illumina sequencing identified 106 DEMs. These DEMs influence endometrium receptivity and embryo implantation by regulating pathways or processes related to endometrial remodeling and immune processes. This is according to the interaction analysis of miRNAs and the target genes, as well as the target gene functional annotation. Taken together, our research provides a basis for further studying the role of miRNAs in goat endometrium receptivity and embryo implantation.

## Figures and Tables

**Table 1:** Summary of sequencing data filtration.

Sample	Total Reads	N% > 10%	5′ Adapter Contaminant	3′ Adapter Null or Insert Null	With Poly A/T/G/C	Low Quality	Clean Reads
C16	16,383,161 ± 1,830,759	347	18,348	651,315	4922	16,774	1,5691,455 ± 1,996,624
	100.00%	0.00%	0.11%	3.98%	0.03%	0.10%	95.78%
P16	15,358,745 ± 2,529,672	351	105,748	1,846,119	51,931	21,706	13,332,890 ± 2,607,147
	100.00%	0.00%	0.69%	12.02%	0.34%	0.14%	86.81%

## Data Availability

The datasets presented in this study can be found in online repositories. The raw reads produced in this study were deposited in the NCBI Sequencing Read Archive. The accession number is PRJNA744709.

## Supplementary Materials

The following are available online at https://www.mdpi.com/article/10.3390/cells10092308/s1, Figure S1: Pearson correlation between samples, Table S1: the information of differentially expressed miRNAs, Table S2: primers used for the miRNAs validation by RT-qPCR, Table S3: target genes of differentially expressed miRNA, Table S4: GO enrichment analysis of the target genes of DEMs, Table S5: KEGG pathway analysis of the target genes of DEMs.
