# Supplementary figures and images for: Differential Expression Pattern of Goat Uterine Fluids Extracellular Vesicles miRNAs during Peri-Implantation
Source: Cells. 2021 Sep 3;10(9):2308. doi: 10.3390/cells10092308 (PMC8470123; doi:10.3390/cells10092308)

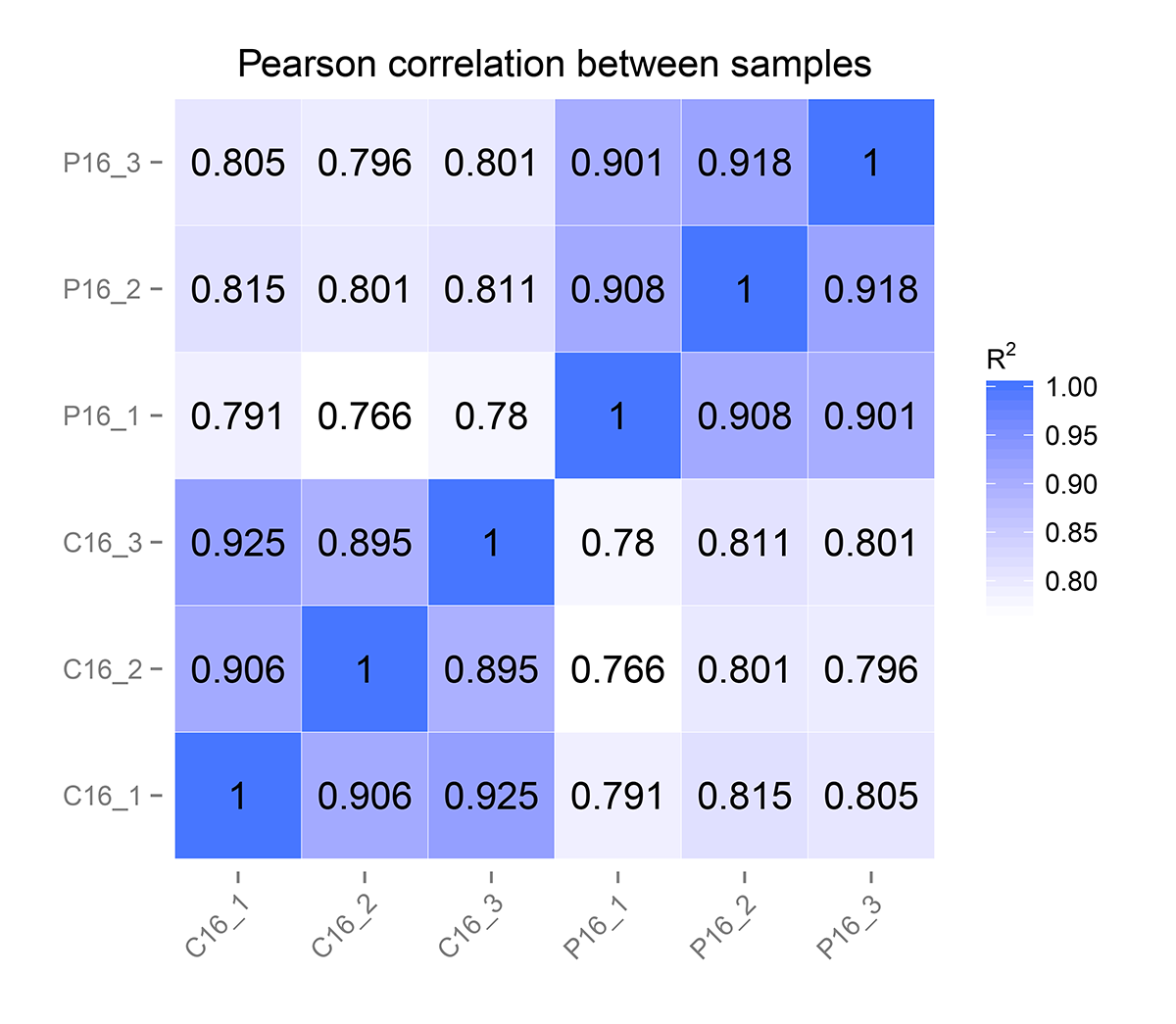

Supplement: Supplementary file 1 [file cells-10-02308-s001.zip › Supplementary Figure S1.tif]
